# Advancements in Cholelithiasis Diagnosis: A Systematic Review of Machine Learning Applications in Imaging Analysis

**DOI:** 10.7759/cureus.66453

**Published:** 2024-08-08

**Authors:** Almegdad S Ahmed, Sharwany S Ahmed, Shakir Mohamed, Noureia E Salman, Abubakr Ali M Humidan, Rami F Ibrahim, Rammah S Salim, Ahmed A Mohamed Elamir, Elmahdi M Hakim

**Affiliations:** 1 Faculty of Medicine, University of Khartoum, Khartoum, SDN; 2 Faculty of Postgraduate Studies, National University - Sudan, Khartoum, SDN; 3 Department of Pediatric Surgery, El-Sahel Teaching Hospital, Cairo, EGY; 4 Faculty of Medicine, Karary University, Khartoum, SDN

**Keywords:** diagnostic models, imaging analysis, machine learning, cholelithiasis, gallstone disease

## Abstract

Gallstone disease is a common condition affecting a substantial number of individuals globally. The risk factors for gallstones include obesity, rapid weight loss, diabetes, and genetic predisposition. Gallstones can lead to serious complications such as calculous cholecystitis, cholangitis, biliary pancreatitis, and an increased risk for gallbladder (GB) cancer. Abdominal ultrasound (US) is the primary diagnostic method due to its affordability and high sensitivity, while computed tomography (CT) and magnetic resonance cholangiopancreatography (MRCP) offer higher sensitivity and specificity. This review assesses the diagnostic accuracy of machine learning (ML) technologies in detecting gallstones.

This systematic review followed the Preferred Reporting Items for Systematic Reviews and Meta-Analyses (PRISMA) guidelines for reporting systematic reviews and meta-analyses. An electronic search was conducted in PubMed, Cochrane Library, Scopus, and Embase, covering literature up to April 2024, focusing on human studies, and including all relevant keywords. Various Boolean operators and Medical Subject Heading (MeSH) terms were used. Additionally, reference lists were manually screened. The review included all study designs and performance indicators but excluded studies not involving artificial intelligence (AI)/ML algorithms, non-imaging diagnostic modalities, microscopic images, other diseases, editorials, commentaries, reviews, and studies with incomplete data. Data extraction covered study characteristics, imaging modalities, ML architectures, training/testing/validation, performance metrics, reference standards, and reported advantages and drawbacks of the diagnostic models.

The electronic search yielded 1,002 records, of which 34 underwent full-text screening, resulting in the inclusion of seven studies. An additional study identified through citation searching brought the total to eight articles. Most studies employed a retrospective cross-sectional design, except for one prospective study. Imaging modalities included ultrasonography (four studies), computed tomography (three studies), and magnetic resonance cholangiopancreatography (one study). Patient numbers ranged from 60 to 2,386, and image numbers ranged from 60 to 17,560 images included in the training, validation, and testing of the diagnostic models. All studies utilized neural networks, predominantly convolutional neural networks (CNNs). Expert radiologists served as the reference standard for image labelling, and model performances were compared against human doctors or other algorithms. Performance indicators such as sensitivity, specificity, positive predictive value (PPV), and negative predictive value (NPV) were commonly used.

In conclusion, while the reviewed machine learning models show promising performance in diagnosing gallstones, significant work remains to be done to ensure their reliability and generalizability across diverse clinical settings. The potential for these models to improve diagnostic accuracy and efficiency is evident, but the careful consideration of their limitations and rigorous validation are essential steps toward their successful integration into clinical practice.

## Introduction and background

The global prevalence of gallstone disease varies between 5% and 20%, affecting millions worldwide [1]. Gallstone disease prevalence varies with age and gender, with females at higher risk due to hormonal influences [2,3]. Progesterone slows gallbladder (GB) emptying, while estrogen promotes cholesterol accumulation, leading to gallstone formation. Other risk factors include obesity, which increases estrogen levels, while rapid weight loss disrupts bile production, also increasing gallstone formation. Also, conditions such as diabetes or anatomical injuries that reduce gallbladder contractions or intestinal motility further increase the risk. Additionally, genetic predisposition plays a role in gallstone susceptibility [4]. Gallstones can become symptomatic causing a condition called calculous cholecystitis, which is commonly treated surgically by cholecystectomy [5]. Furthermore, gallstones can move to the common bile duct causing serious complications such as cholangitis, biliary pancreatitis, and gallstone ileus and increasing the risk for gallbladder cancer significantly [6-8].

The prompt diagnosis and treatment of gallstones are important to avoid complications, with abdominal ultrasound (US) commonly being the initial diagnostic method to be applied [9]. US is simple, relatively affordable, and practical and has high sensitivity in detecting gallstones [10]. Other methods included computed tomography (CT) scans; compared to US, reports showed that CT has better sensitivity and specificity in diagnosing gallstones [11,12]. However, it also poses an increased risk of radiation and has higher costs than US [9]. Magnetic resonance cholangiopancreatography (MRCP) has high sensitivity and specificity in diagnosing gallstones; however, it is less effective with smaller gallstones of less than 3 mm [13].

The applications of machine learning (ML) in healthcare have seen massive advancements, with technologies being applied successfully in image analysis in the fields of pathology, radiology, and dermatology [5]. The integration of machine learning technology in gallstone disease diagnosis could be beneficial in increasing the efficiency of the diagnosis, reducing costs, and decreasing the workload on healthcare professionals. However, with the emergence of new technologies comes the necessity of rigorous testing in terms of efficacy and cost-effectiveness. The aim of this review was to evaluate the diagnostic accuracy of machine learning technologies applied in the diagnosis of cholelithiasis.

## Review

Methods

This systematic review was conducted in compliance with the guidelines of Preferred Reporting Items for Systematic Reviews and Meta-Analyses (PRISMA) [14]. The study aimed primarily to answer the following question: what is the accuracy of machine learning technologies that are used in the detection of gallstones? Secondary endpoints included answering the following: What architectures are used in the development of these technologies? What are the advantages and drawbacks of these technologies? To do that, we performed an electronic search in four databases: PubMed, Cochrane Library, Scopus, and Embase.

The search covered published literature up to April 2024, restricted to human studies and English language records. Search keywords included “cholelithiasis,” “gallstones,” “biliary calculi,” “gallbladder stones,” “artificial intelligence,” “machine learning,” “deep learning,” “neural networks,” “computer-aided diagnosis,” “image analysis,” “image classification,” “pattern recognition,” “automatic segmentation,” “entropy degradation method,” and “partially observable Markov decision process.” The search process was performed using Medical Subject Heading (MeSH) terms and using various Boolean operators. Additionally, the reference lists of relevant studies were manually screened for any potential included studies.

The records of all study designs and populations were considered for this review, excluding editorials, commentaries, reviews, and studies having incomplete data regarding the performance of the algorithms. Additionally, all performance indicators were considered for the purpose of the assessment. We also excluded studies that used non-artificial intelligence (AI)/machine learning algorithms for image segmentation, studies that used non-imaging diagnostic modalities, studies that used microscopic images or histopathology, and studies that assessed other diseases.

After the selection of the studies, relevant information was extracted. The information includes basic study characteristics such as design, country, the number of participants, imaging modalities used, and the number of images used. Additionally, we collected information about machine learning architectures and algorithms used, training, testing, and validation for the diagnostic models. We also extracted information regarding the performance of these diagnostic models according to the performance indicators used in the included study and the reference standards and comparison diagnostics against which they were evaluated. Furthermore, we recorded information about the advantages and drawbacks of each diagnostic model as they were reported in the included studies.

As for the risk of bias assessment, the Quality Assessment of Diagnostic Accuracy Studies-2 (QUADAS-2) tool was used, which is a tool used for the assessment of the risk of bias and applicability in systematic reviews of primary diagnostic test accuracy studies [15]. The tool assesses four domains: patient selection, index test, reference standard, and flow and timing. The tool uses signalling questions in the assessment process.

Results 

Study Selection and the Characteristics of the Included Studies

**Figure 1:**
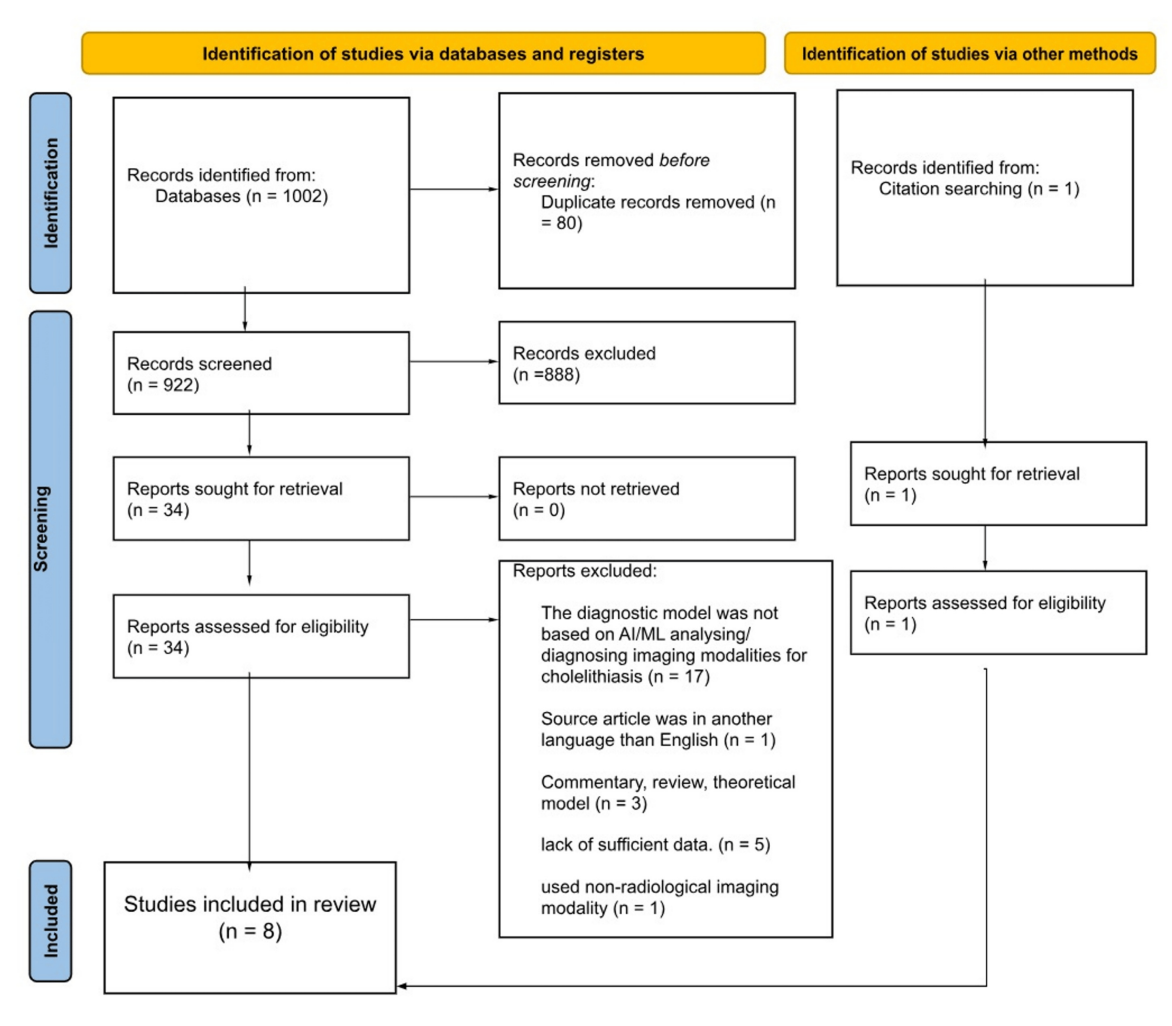
PRISMA flowchart of the study selection process PRISMA, Preferred Reporting Items for Systematic Reviews and Meta-Analyses; AI, artificial intelligence; ML, machine learning

The electronic literature search identified 1,002 records from four databases; after the title and abstract screening phase, 34 records were subjected to full-text screening leaving seven articles to be included in the review. Furthermore, after citation searching for the relevant records, an additional article was added raising the total of the included articles to eight; the full process of the selection of these articles is illustrated in Figure 1.

Most of the included studies followed a retrospective cross-sectional design with the exception of one study in which a prospective cross-sectional design was used [16]. As for the imaging modalities used in the studies, four studies utilized ultrasonography, and three of them used computed tomography (CT), with only one study using magnetic resonance cholangiopancreatography (MRCP) [17]. The number of patients and images used in these studies varied, ranging from 60 to 2,386 patients and from 60 to 17,560 images. Table 1 shows the characteristics of the included studies. Performance was evaluated based on accuracy, sensitivity, specificity, mean average precision (mAP), precision, and recall. Reliability was evaluated according to intersection over union (IoU) and F1 score.

**Table 1:** Characteristics of the included studies CT, computed tomography; US, ultrasound; MRCP, magnetic resonance cholangiopancreatography; EUS, endoscopic ultrasound

Study	Country	Study design	Number of patients/images	Imaging modality
Logeswaran, 2006 [17]	Malaysia	Retrospective cross-sectional	593 images (105 used for training and 488 for validation)	MRCP
Lian et al., 2017 [18]	China	Retrospective cross-sectional	60 patients (60 images)	US
Pang et al., 2019 [19]	China	Retrospective cross-sectional	1,369 patients (total of 5,986 images: 4,000 used for training, 986 for validation, and 1,000 for testing)	CT
Pang et al., 2019 [16]	China	Prospective cross-sectional	100 patients (total of 1,300 images: 673 used for training and 627 for verification)	CT
Song et al., 2019 [20]	China	Retrospective cross-sectional	196 patients (a total of 5,350 images: 4,500 used for training, 350 for validation, and 500 for testing)	CT
Jang et al., 2021 [21]	South Korea	Retrospective cross-sectional	753 patients (1,122 images)	EUS
Yu et al., 2021 [22]	Taiwan	Retrospective cross-sectional	2,386 patients (a total of 17,560 images: 14,048 used for training, 1,756 for validation, and 1,756 for testing)	US
Veena et al., 2022 [23]	India	Retrospective cross-sectional	60 patients (a total of 60 images: 48 used for training and 12 for testing)	US

All the included studies used neural networks, with convolutional neural networks (CNNs) being the most commonly used technology. The reference standard used by all the studies was expert doctors labelling the used images. As for the comparisons, the developed models were compared to either human experts or other algorithms. Furthermore, the included studies utilized various performance indicators to assess their diagnostic models, with parameters such as sensitivity, specificity, positive predictive value (PPV), and negative predictive value (NPV) as the most commonly used ones. Table 2 shows the characteristics of the diagnostic models of the included studies.

**Table 2:** Characteristics of diagnostic models in included studies ANN, artificial neural network; CNN, convolution neural network; PA-PCNN, parameter-adaptive pulse-coupled neural network; NPV, negative predictive value; EVA, average similarity percent of contours; mAP, mean average precision; IoU, intersection over union; PPV, positive predictive value; AUROC, area under the receiver operating characteristic; MCC, Matthew’s correlation coefficient; GVF, gradient vector field; FCM, fuzzy c-means; LB-FCM, learning-based fuzzy c-means; SSD, single-shot multibox detection; R-CNN, region-based convolution neural network; YOLO, you only look once; FPN, feature pyramid network

Study	ML architecture	Reference standard	Comparison	Performance indicator(s)
Logeswaran, 2006 [17]	ANN	Expert radiologists	Expert radiologists	Sensitivity, specificity, overall accuracy, and NPV
Lian et al., 2017 [18]	PA-PCNN	Expert radiologists	Other models (snake GVF {SG}, snake distance {SD}, and snake balloon {SB} algorithms)	EVA and runtime
Pang et al., 2019 [19]	CNN	Expert radiologists	Other models (general YOLOv3 model)	mAP
Pang et al., 2019 [16]	CNN	Expert radiologists	Other models (SSD300, SSD512, YOLOv2, and MobileNetV1 algorithms)	Average accuracy rate
Song et al., 2019 [20]	CNN	Expert radiologists	Other models (previous model {U-Net}, FCM, and LB-FCM algorithms)	IoU
Jang et al., 2021 [21]	CNN	Endoscopists	Endoscopists	Sensitivity, specificity, PPV, NPV, accuracy, and AUROC
Yu et al., 2021 [22]	CNN	Expert radiologists	Other models (Faster R-CNN + ResNet-50, Faster R-CNN + Inception V2, SSD-FPN + MobileNetV1, SSD + Inception V2, and SSD + MobileNetV2 algorithms)	Sensitivity, specificity, precision, recall, F1 score, MCC, inference speed, AUROC, and average precision
Veena et al., 2022 [23]	CNN	Expert radiologists	Other models (SSD, Faster R-CNN, and Mask R-CNN algorithms)	Precision, recall, mAP, and F1 score

Risk of Bias Assessment

The risk of bias of included studies was assessed using the QUADAS-2 tool. This tool evaluates four key domains: patient selection, index test, reference standard, and flow and timing. The results are summarized in Table 3. The assessment identified studies with varying risks of bias. As for the patient selection domain, most of the studies exhibited unclear risk of bias; this uncertainty mainly comes in the area of using prospective or consecutive methods of selecting patients’ images in the datasets of training, testing, and validation of machine learning models. Regarding the index test domain, most of the studies showed low risks of bias with the exception of one study that showed unclear risk of bias [18]. All studies showed a low risk of bias in the reference standard and flow and timing domains. Additionally, there were low concerns regarding the applicability of the included studies to the review across all domains.

**Table 3:** Risk of bias and applicability assessment results for included studies

Study	Risk of bias				Applicability concerns		
	Patient selection	Index test	Reference standard	Flow and timing	Patient selection	Index test	Reference standard
Logeswaran, 2006 [17]	Low risk	Low risk	Low risk	Low risk	Low concern	Low concern	Low concern
Lian et al., 2017 [18]	Unclear risk	Unclear risk	Low risk	Low risk	Low concern	Low concern	Low concern
Pang et al., 2019 [19]	High risk	Low risk	Low risk	Low risk	Low concern	Low concern	Low concern
Pang et al., 2019 [16]	Low risk	Low risk	Low risk	Low risk	Low concern	Low concern	Low concern
Song et al., 2019 [20]	Unclear risk	Low risk	Low risk	Low risk	Low concern	Low concern	Low concern
Jang et al., 2021 [21]	Unclear risk	Low risk	Low risk	Low risk	Low concern	Low concern	Low concern
Yu et al., 2021 [22]	Unclear risk	Low risk	Low risk	Low risk	Low concern	Low concern	Low concern
Veena et al., 2022 [23]	Unclear risk	Low risk	Low risk	Low risk	Low concern	Low concern	Low concern

Performance of the Diagnostic Models

The included studies used different sorts of imaging modalities, with four of them using ultrasonography. The study by Lian et al. utilized 60 grey-level ultrasound images to develop a diagnostic model based on a parameter-adaptive pulse-coupled neural network (PA-PCNN). This study emphasized a modified preprocessing step to enhance image quality prior to classification using the modified Otsu method and modified anisotropic diffusion methods. This was followed by obtaining the fine segmentation of the gallbladder using a morphology filtering algorithm; then, PA-PCNN was used to record coarse segmentation, and lastly, the final segmentation results were obtained using the locally estimated scatterplot smoothing (LOESS) algorithm (locally weighted regression smoothing). The reference standard comprised assessments by two expert radiologists. The experimental results indicated that the model achieved high accuracy in detecting ultrasound image features, with an average similarity percent of contours (EVA) of 79.81% and a runtime of 0.66 seconds. The primary advantage of this method lies in its ability to automatically perform image segmentation and feature extraction, thereby minimizing manual intervention and enhancing the consistency of image analysis [18].

Jang et al. conducted a study using a significant number of endoscopic ultrasound (EUS) images to develop their machine learning model, totaling 1,122 images. Their main goal was the differentiation between gallstones and GB polyps based on the EUS images, indirectly contributing to identifying gallstones. They utilized a deep learning architecture called ResNet-50 architecture, which is a CNN model specifically tailored for ultrasound image analysis. ResNet-50 addresses the challenge of vanishing gradients, a common obstacle in deep neural networks, by incorporating residual connections. This technique facilitates the training of deeper architectures by allowing the network to learn residual functions that map the input to the desired output. Furthermore, the model is initialized with pretrained weights obtained from the ImageNet dataset [21].

The reference standard in Jang et al.’s study was established by comparing the model’s performance to the evaluations of endoscopists. The training dataset included various types of gallstone disease including extrahepatic choledocholithiasis, cholecystolithiasis, and intrahepatic choledocholithiasis. The model demonstrated high accuracy in identifying and categorizing ultrasound images, with a sensitivity of 98.4% and specificity of 84.2% for the model, compared to 74.7% and 97.9% for the endoscopists, respectively. Additionally, the overall accuracy of the model was 95.7%, compared to 94.9% for the endoscopists. Furthermore, the PPV and NPV for the model were 96.3% and 92.9%, compared to 86.1% and 96.2% for the endoscopists, respectively. It is clear that the model had higher overall accuracy, sensitivity, and PPV compared to the endoscopists; however, it had a lower specificity and NPV. The key advantage reported was the model’s ability to process images rapidly while maintaining high diagnostic accuracy. With more advancements in technology and training, the model can perhaps be able to analyze EUS videos in real time. This has been one of the limitations of this model since using representative EUS images may potentially cause image distortions, leading to exaggerating or missing potential findings compared to real-time EUS observations. Another limitation of the model was the use of lesions with a maximum diameter of 7-20 mm and the exclusion of smaller lesions, which may limit the generalizability of the EUS-AI system in clinical practice [21].

Yu et al. also focused on the use of ultrasound images, conducting a study utilizing CNN architectures to develop machine learning models for the automated detection of gallstones and cholecystitis using abdominal ultrasound images. Specifically, the study employed single-shot multibox detection (SSD) and feature pyramid network (FPN) algorithms integrated with ResNet-50 for gallstone detection and MobileNetV2 for cholecystitis detection. These two models were fine-tuned from pretrained CNN models, enhancing their ability to accurately identify relevant features in ultrasound images [22].

The fine-tuning process involved further training the pretrained models on the specific ultrasound datasets. The performance of the fine-tuned CNN models was evaluated against other models such as Faster region-based CNN (R-CNN) with ResNet-50 and Inception V2, SSD-FPN with MobileNetV1, SSD with Inception V2, and MobileNetV2. The reference standard for comparison was established by two expert radiologists. For gallstone detection, the SSD-FPN-ResNet-50 model achieved an average precision of 86.95%, ranking third among the models tested, with Faster R-CNN-ResNet-50 and Faster R-CNN-Inception V2 leading slightly ahead. The model also demonstrated high sensitivity (92%), precision (93%), and an F1 score of 92% while maintaining an inference speed of 21 ms, also ranking third in detection time [22].

This study highlighted several advantages and drawbacks of the developed model. One significant advantage is the model’s potential to mitigate the operator-dependent accuracy of point-of-care ultrasound, thereby potentially reducing emergency department lengths of stay and supporting early cholecystectomy. However, the study also acknowledged several limitations, including the incomplete visibility of the gallbladder in some images, which could affect detection accuracy. The reliance on still images rather than moving ones might limit the model’s ability to differentiate between small gallstones and gallbladder polyps. Furthermore, the study’s use of data from only two types of ultrasound machines could impact the model’s robustness, as real-world practice involves variability in image quality from different machines. Despite preprocessing efforts, this variability might not be fully captured, potentially affecting performance [22].

Veena et al.’s study also utilized ultrasound images, employing CNNs. Their research utilized object detection models, including SSD, Faster R-CNN, and Mask R-CNN, to improve the precision of gallstone detection. The models were trained using preprocessing and augmentation techniques to enhance the quality and diversity of the training dataset. The study aimed to develop an integrated system capable of predicting the presence of gallstones and visualizing these predictions through a web application developed with the Streamlit framework. The reference standard for model evaluation involved annotations provided by expert radiologists, and the performance of the diagnostic models was compared against each other [23].

The findings of Veena et al.’s study indicated that the Mask R-CNN model, particularly when using the ResNet-101-FPN backbone network combination, demonstrated superior performance in object detection tasks. While it exhibited a slightly lower precision compared to the Faster R-CNN model (0.78 versus 0.867), Mask R-CNN outperformed the other models in terms of recall, mAP, and F1 score. However, the study also acknowledged several limitations. The complexity and computational demands of the models require powerful hardware resources, and the extensive training data requirements pose challenges in data collection and annotation. The study utilized a relatively small dataset, potentially limiting the generalizability of the models. Furthermore, the manual annotation of images with bounding boxes or segmentation masks required the expertise of medical professionals, making the process resource-intensive and prone to human error. The study also noted a lack of detailed performance metrics and comprehensive comparisons with other existing models in the literature, which could provide further insights into the proposed approach’s effectiveness [23].

Three of the included used CT images in developing their diagnostic models. Pang et al. conducted a study using the you only look once version 3 (YOLOv3) framework, a fully convolutional network (FCN) with 75 convolutional layers, for the detection and classification of gallstones in CT images. YOLOv3 is known for its efficiency and speed, employing skip connections and up-sampling layers instead of pooling to preserve low-level features. This performance, coupled with its ability to process approximately 200 CT images in about four seconds, makes it significantly faster than traditional methods. The study enhanced detection accuracy by focusing on five key objects: the spine, liver, gallbladder, granular gallstones, and muddy gallstones, leveraging their stable location information [19].

The YOLOv3 model’s bounding box predictions and class scores were refined using specific strategies, such as adjusting confidence levels based on the simultaneous identification of the liver and gallbladder. This approach resulted in high detection accuracy, with the model achieving over 95% recognition accuracy for the liver and gallbladder, 98% for granular gallstones (with an average of 92.7%), and 87% for muddy gallstones (with an average of 80.3%). The overall average detection accuracy for gallstones was 86.5%. Compared to the general YOLOv3 model, this new model (YOLOv3-arch) demonstrated improvements of 3.5% and 8% in identifying granular and muddy gallstones, respectively. The significant speed advantage of the model highlights its potential to save considerable time for medical professionals in diagnosing gallstones [19].

Another study performed by Song et al. introduced an approach to gallstone segmentation in CT images by developing the U-NeXt model, an enhancement of the traditional U-Net CNN. The U-NeXt model incorporates an attention mechanism and deep aggregation, which significantly improves the training effect and segmentation accuracy. This architecture includes dense skip connections and nested connections, allowing for multi-scale feature generation. Dense connections throughout the model facilitate the extraction of more detailed features. The performance of U-NeXt was evaluated against several other image segmentation methods such as U-Net, fuzzy c-means (FCM), and learning-based (LB)-FCM. U-NeXt demonstrated an improvement in gallstone characterization, outperforming U-Net by approximately 7.03%, U-Net with Res-Blocks by 8.33%, LB-FCM by 24.34%, and FCM by 29.7%. The evaluation metric used was IoU [20].

The study highlighted two main advantages of this model, the up-sampling module with an attention mechanism, which enhances the weight of useful information, and the skip-spatial pyramid pooling (SPP) module, which fuses more multi-scale information than the ordinary SPP module, thereby improving segmentation accuracy. However, the study also noted some limitations. The dataset consisted solely of images from patients with cholelithiasis, lacking control subjects without gallstones. Additionally, the absence of independent external validation means that the generalizability of the algorithm to other clinical settings remains uncertain [20].

Another study was also performed using CT images, in which Pang et al. introduced a lightweight convolutional neural network-based diagnostic system designed to operate on a mobile Android platform. This system begins with image preprocessing techniques such as histogram equalization and nonlinear stretching to enhance image contrast, followed by the labelling of regions of interest (ROI) on the CT images. The model utilizes the MobileNetV2 architecture, known for its efficiency and low computational demands, to extract features and discern gallstones. After processing the ROI through the neural network, the system generates an end-to-end-labelled image highlighting the presence of cholelithiasis, along with an electronic medical report detailing the user’s information, number and size of gallstones, and medical advice [16].

The performance of this lightweight CNN was benchmarked against other well-known algorithms, including SSD300, SSD512, YOLOv2, and MobileNetV1. The system demonstrated a recognition accuracy rate of approximately 90.8%, which is competitive with SSD300 (92.0%), SSD512 (91.8%), YOLOv2 (90.7%), and MobileNetV1 (92.4%). Additionally, the system’s speed is noteworthy, completing the recognition process in less than four seconds. The system’s adaptability to different working environments enhances its practical utility in various clinical settings [16].

As for MRCP, only one study assessed the performance of a diagnostic model using this technique. Logeswaran (2006) employed an artificial neural network (ANN) for the detection of biliary stones. The algorithm used for image segmentation was the watershed algorithm, which effectively delineates regions in the image. To detect the biliary structure, a segment-based region-growing strategy was applied, enhancing control over the detection mechanism in the presence of noisy and complex images typical of MRCP scans. Following segmentation, a supervised feedforward ANN was utilized to detect stones within the biliary tract. The performance of the system was evaluated against radiologists as a reference standard. The algorithm correctly identified 90 true-positive images out of 132 and 316 true-negative images out of 461. The system achieved a sensitivity of 68.18%, a specificity of 68.55%, an overall accuracy of 68.47%, and an NPV of 88.3% [17].

Several advantages were noted in this model, including the ability to diagnose stones using only a single 2D MRCP thick slab image, which simplifies the diagnostic process. However, the system’s performance was limited by several factors, such as the presence of high-intensity background tissue, folds, and non-uniform intensity within the biliary structure. These issues can obscure the detection of stones, particularly high-intensity stones or those located adjacent to low-intensity walls, affecting the algorithm’s accuracy [17].

Discussion

This systematic review synthesized findings from eight studies that evaluated the performance of machine learning models in diagnosing gallstones using various imaging modalities. Neural networks, particularly CNNs, were the cornerstone of the diagnostic models across all studies. Advanced architectures such as YOLOv3, U-NeXt, MobileNetV2, and ResNet-50 were employed, demonstrating the adaptability and effectiveness of these models in medical image analysis. The reference standard for model evaluation was consistently based on expert radiologist assessments. The models demonstrated substantial variability in performance, with most of them achieving high detection accuracy with notable speed.

Several advantages were highlighted in the reviewed studies. Models such as U-NeXt and YOLOv3 were praised for their innovative use of attention mechanisms and skip connections, which enhanced feature extraction and segmentation accuracy. The rapid processing capabilities of models such as MobileNetV2 suggest the potential for real-time application in clinical settings, potentially reducing diagnostic times and improving patient outcomes. Additionally, the automated nature of these models can minimize manual intervention, thus enhancing the consistency and reproducibility of image analysis.

The studies also identified significant limitations. The generalizability of the models was often constrained by the homogeneity of the datasets, particularly the lack of control subjects, the reliance on images from limited types of ultrasound machines, and the lack of external validation in some studies. This limitation raises concerns about the models’ robustness across different clinical environments and imaging equipment. Furthermore, technical challenges such as the presence of high-intensity background tissue, non-uniform intensity, and image artifacts were noted to affect model performance, particularly in the segmentation tasks. Additionally, the majority of the included studies employed retrospective cross-sectional designs, with only one study adopting a prospective approach. The computational demands of complex models such as Mask R-CNN also pose practical challenges, necessitating powerful hardware and extensive training data, which may not always be feasible. The manual annotation required for training these models, involving expert radiologists, is another resource-intensive aspect that could limit scalability.

Some limitations are also due to the nature of gallstones diagnosis; for example, it has been reported that the differentiation of gallstones from polyps becomes less reliable for lesions under 5 mm [24-26]. However, the diagnosis of larger stones is crucial since the presence of gallstones larger than 3 cm is associated with a 10% increase in the likelihood of gallbladder cancer compared to gallstones smaller than 1 cm [7]. The significance of these models is amplified by the diagnostic challenge of differentiating early-stage gallbladder carcinoma, which frequently presents with nonspecific symptoms that mimic those of cholelithiasis, and here might come the role of these models [27,28].

Future research should focus on addressing these limitations by incorporating more diverse and extensive datasets, including control subjects, and conducting independent external validations to ensure broader applicability. Exploring real-time analysis capabilities and integrating these models into routine clinical workflows could enhance their practical utility. Additionally, continued collaboration between radiologists and data scientists is crucial to refine these models and address any persisting challenges.

## Conclusions

While the reviewed machine learning models show promising performance in diagnosing gallstones, significant work remains to be done to ensure their reliability and generalizability across diverse clinical settings. The potential for these models to improve diagnostic accuracy and efficiency is evident, but the careful consideration of their limitations and rigorous validation are essential steps toward their successful integration into clinical practice.
